# Case report: Cardiac arrest during carotid body tumor resection indicating carotid sinus hypersensitivity

**DOI:** 10.3389/fcvm.2022.996644

**Published:** 2022-12-16

**Authors:** Hong Yong Duan, Qiang Guan, Yu Jie Guo, Ning Liang

**Affiliations:** ^1^Department of Vascular Surgery, Shanxi Provincial People's Hospital, Taiyuan, China; ^2^Department of Vascular Surgery, The Fifth Clinical Medical College of Shanxi Medical University, Taiyuan, China

**Keywords:** carotid body tumors, carotid sinus hypersensitivity, intraoperative cardiac arrest, surgical complications, tumor resection

## Abstract

**Background:**

Carotid body tumor surgery is associated with various complications. However, intraoperative cardiac arrest is very rare and no more than 10 cases have been reported.

**Case description:**

A 58-year-old woman diagnosed with bilateral carotid body tumors underwent right carotid body tumor surgery. Sudden cardiac arrest occurred during the resection and was attributed to carotid sinus hypersensitivity. The patient recovered after prompt treatment and the tumor was removed completely with no complications.

**Conclusion:**

Cardiac arrest attributed to carotid sinus hypersensitivity during carotid body tumor resection is very rare. Proper treatments can reverse intraoperative cardiac arrest. If carotid sinus hypersensitivity is detected preoperatively, prophylactic temporary pacemaker implantation may be appropriate.

## Introduction

Carotid body tumors (CBTs) are rare neurogenic tumors that arise from chemoreceptive tissue in the carotid body. It is the most common form of neck paragangliomas. Although there is no effective drug to treat CBT, several studies have shown that close monitoring and follow-up are an option in select CBT patients who were asymptomatic, showed no signs of accelerated enlargement, and presented with no evidence of malignancy or no somatic mutation (1–3). While complete surgical resection remains the mainstay of CBT treatment and the only definitive cure for CBT (4), tumor manipulation is associated with significant morbidity due to the nature of its anatomic structure and location. The rate of surgery-related complications has been reported to be in the range from 20 to 27% (5). Herein, we report on a female patient with bilateral CBTs who underwent surgical manipulation complicated by cardiac arrest during her first staged tumor resection. The clinical presentation of hemodynamic instability indicated carotid sinus hypersensitivity (CSH). Case details are provided and the related literature is comprehensively reviewed.

## Case report

**Figure 1:**
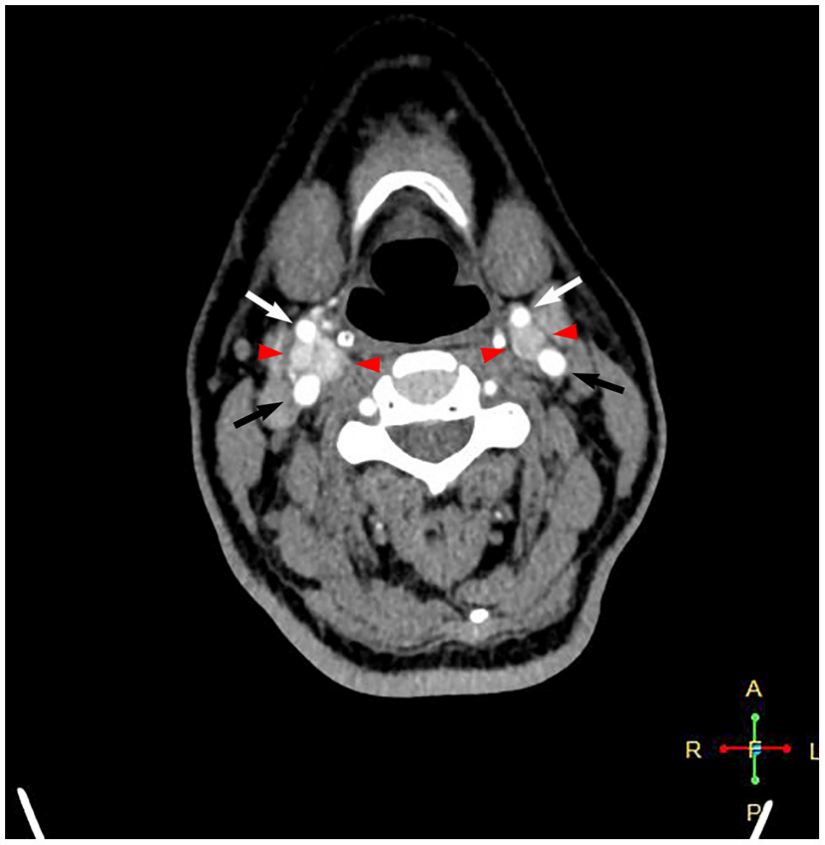
Computed tomography angiography showing intensely enhanced masses at the level of bilateral carotid bifurcations. Black arrows point to the internal carotid artery, white arrows point to the external carotid artery, and red arrowheads point to the carotid body tumor.

A 58-year-old female patient who was diagnosed with bilateral CBTs during a routine health check-up 3 years prior was admitted to the department of vascular surgery, Shanxi Provincial People's Hospital. She complained of a swelling sensation in her neck. The patient had no local pain, difficulty swallowing or breathing, voice changes, or palpitations. No systemic symptoms, such as fever, chills, and malaise, were present. There were no special records in her past medical history and family history. During a physical examination, a small, painless, firm, and pulseless mass was palpated on the right side of the neck at the carotid triangle located below the angle of the mandible. The Fontaine sign was positive. Physical examination did not reveal a left neck mass. The laboratory analyses did not suggest any other abnormalities, except slightly elevated low-density lipoprotein cholesterol and triglyceride levels. A routine electrocardiogram was normal. A computed tomographic angiography showed an intensely enhanced mass measuring 2.6 × 1.8 × 1.3 cm at the level of the right carotid bifurcation. A smaller mass measuring 1.1 × 0.9 × 0.9 cm was present on the left in a similar location (Figure 1). These findings were consistent with bilateral CBTs. According to the imaging results, the right tumor partially surrounded the carotid vessels, indicating Shambling II group tumor. The left-side tumor was of Shambling I group.

The therapy options were discussed with the patient after admission. She agreed to staged surgical dissection of the tumors. According to most experts' consensus, the smaller tumor should be removed first because of the low operative risk for permanent cranial nerve injury. This strategy facilitates the resection of large tumors in the second stage. However, the risk of manipulation in the right CBT was as low as that in the left after evaluation, and the patient requested removal of the large tumor first. Therefore, it was decided that the right CBT was to be resected in the first stage. The patient was informed of the possible surgical complications, and written consent was obtained before surgery.

**Figure 2:**
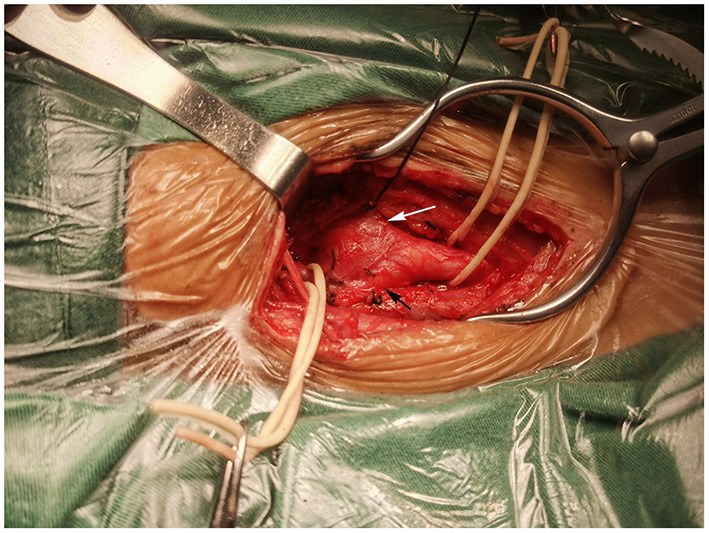
Intraoperation view of right carotid body tumor resection. A mass at the bifurcation splayed the internal carotid artery (black arrow) and external carotid artery (white arrow).

**Figure 3:**
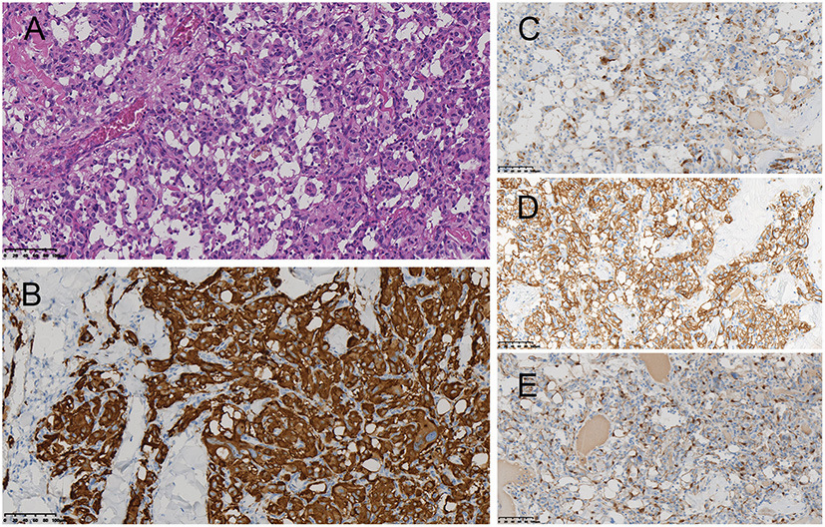
Histopathological examination revealed the zellballen pattern characteristic of paraganglioma **(A)**. Immunohistochemical results were positive for synaptophysin **(B)**, chromogranin A **(C)**, and CD56 **(D)** in the tumor cells and S-100 protein in the supporting cells **(E)**.

Surgery was performed in a supine position under general orotracheal anesthesia, and the neck was slightly hyperextended and rotated to the left. A longitudinal incision along the anterior border of the right sternocleidomastoid muscle was made to expose the carotid bifurcation and the tumor (Figure 2). Proximal and distal arterial control was obtained by exposing the common carotid artery, internal carotid artery, and external carotid artery cephalad to the mass. Then, the tumor was gradually separated from the carotid arteries in cranial to caudal direction along the subadventitial plane using bipolar electrocautery. Cardiac arrest suddenly occurred when dissecting the tumor from the carotid bifurcation. The systolic blood pressure (sBP) dropped from 115 to 40 mmHg. This complication was managed immediately with external cardiac massage and administration of a single intravenous 0.5-mg atropine dose while simultaneously suspending CBT manipulation. The cardiac activity recovered ~40 s after asystole. The heart rate and sBP returned to 72–80 bpm and 120 mmHg, respectively. The manipulation was then completed while gently assisted by topical anesthesia with 2% lidocaine. The patient was closely monitored after surgery and a decrease in the heart rate and blood pressure level was not noted. On the second day after surgery, the patient complained of left chest pain during exercise. Chest X-ray showed no rib fracture, and reexamination of the electrocardiogram indicated normal results. The level of myocardial enzymes was not elevated. The pain was relieved over time without analgesia treatment. The patient had no neurological sequelae or cranial nerve injuries after surgery and was discharged 1 week after surgery. Histopathological examination revealed a zellballen pattern characteristic of paraganglioma and did not show any evidence of malignancy (Figure 3A). Immunohistochemical results were positive for synaptophysin, chromogranin A, and CD56 in the tumor cells and S-100 protein was present in the sustentacular cells (Figures 3B–E). Both synaptophysin and chromogranin A are neuroendocrine markers located in neuroendocrine granules. CD56, known as a neuronal cell adhesion molecule, is a membrane glycoprotein present on the neural cell surface. These positive immunohistochemical stain profiles supported the diagnosis of CBT. The patient had no complaints at 1-month follow-up after surgery.

## Discussion

CBTs can be divided into sporadic or familial types. Sporadic types account for the majority of cases, and bilateral tumors are detectable in only 5% of sporadic CBTs (6). A complete staged resection is suitable for most bilateral CBTs. Surgical CBT management can lead to various complications, including cranial nerve injury, vascular injury, stroke, and intraoperative blood loss. However, sudden cardiac arrest during CBT manipulations experienced by the patient described in the present report is very rare, with no more than 10 cases reported to date (7, 8). This complication can be attributed to CSH.

The carotid sinus is a baroreceptor with neurovascular structure located above the carotid bifurcation (9). CSH is an exaggerated response of the carotid sinus baroreceptor to local mechanical stimulation. According to the European Society of Cardiology (10), CSH is defined as asystole lasting for at least 3 s and/or a drop in sBP of at least 50 mmHg in response to carotid sinus massage. The prevalence of CSH is mainly concentrated in people over 60 years old and increases with age (11). It is rare in individuals younger than 50 years old. CSH is divided into three subtypes: (1) the cardioinhibitory type is characterized by a decrease in heart rate that leads to cardiac arrest; (2) the vasodepressor type results in a drop in sBP independent of heart rate; and (3) the mixed type has both features (12). The patient's CSH fell into the third category. CSH most commonly occurs in older males, probably owing to atherosclerotic vascular non-compliance and sternocleidomastoid denervation (11). Many systematic comorbidities and local morbidities are also associated with CSH. The former includes Alzheimer's disease, dementia with Lewy bodies, Takayasu arteritis, severe coronary artery disease, and internal carotid artery occlusion. The latter includes head and neck malignancy, scar in the neck, and radiotherapy to the neck (13, 14).

The study patient had no morbidities associated with CSH mentioned above. Special positioning necessary to obtain adequate tumor exposure might be a CSH trigger. Stretched carotid sinus and vagus nerve caused by hyperextension and rotation of the neck can led to a slowdown of the heart rate. Intraoperative dissection of the tumor from the carotid bifurcation and carotid arteries stimulated the carotid sinus, which was a response to the vessel wall deformation in any direction (15). These stimuli were carried out *via* the glossopharyngeal and vagus nerves to the nucleus tractus solitarius in the brainstem, with the efferent reflex arch carried through the vagus and sympathetic nerves to the heart and blood vessels, negatively influencing the heart rate and blood pressure (11). It is likely that the above two processes, especially the second one, were the main causes of CSH in the study patient.

The incidence of complications mentioned above increases with the expansion of the tumor (16). Intraoperative cardiac arrest seems to be not associated with tumor size. Prophylactic local anesthetic injection around the carotid sinus during the carotid bifurcation operation is routinely used by surgeons to inhibit the carotid sinus reflex (17, 18). However, the effect is questionable. Part of the causation may be due to the variations in carotid sinus location, which results in the inaccurate local anesthesia injection site (9).

The first staged surgery for the right CBT induced CSH in the present patient. The left CBT was smaller in size. The patient decided not to remove it for the time being, but to observe it instead. However, the patient provided consent for removing the tumor on the left side in the future, if necessary. How to prevent possible intraoperative CSH is a problem worthy of discussion. Bilateral CSH has been reported and atropine does not reverse bradycardia every time (19, 20). Temporary cardiac pacemaker implantation is a prophylactic option for CSH detected before surgery (21, 22). The patient might need a temporary pacemaker before the surgery to remove the left CBT.

## Conclusion

Intraoperative cardiac arrest caused by CSH is a very rare but life-threatening complication during the resection of CBT. Its incidence has little correlation with tumor size. Doctors should be alert to this potentially dangerous complication when performing surgery near the carotid sinus. Implantation of a temporary pacemaker is a possible preventive measure if CSH is detected on the surgical side prior to the operation.

## Data availability statement

The original contributions presented in the study are included in the article/supplementary material, further inquiries can be directed to the corresponding author.

## Ethics statement

The studies involving human participants were reviewed and approved by Ethics Committee of Shanxi Provincial People's Hospital. The patients/participants provided their written informed consent to participate in this study. Written informed consent was obtained from the individual(s) for the publication of any potentially identifiable images or data included in this article.

## Author contributions

HYD made contributions to the conception, design of the work, acquisition, analysis, and interpretation of data and has drafted the manuscript. QG made contributions to the manuscript revision. YJG made contributions to the pictures collection. NL made contributions to the pictures editing. All authors approved the submitted manuscript for publication.

## References

[1] Rodriguez-Cuevas S Lopez-Garza J Labastida-Almendaro S. Carotid body tumors in inhabitants of altitudes higher than 2000 meters above sea level. Head Neck. (1998) 20:374–8. 10.1002/(sici)1097-0347(199808)20:5<374::aid-hed3>3.0.co;2-v 9663663

[2] Langerman A Athavale SM Rangarajan SV Sinard RJ Netterville JL. Natural history of cervical paragangliomas: outcomes of observation of 43 patients. Arch Otolaryngol Head Neck Surg. (2012) 138:341–5. 10.1001/archoto.2012.37 22431860

[3] Reitz K Ramos A Speranza G Chaer R Singh M Snyderman C. Non-functional carotid body tumors in patients without somatic mutations may be considered for non-operative management. Ann Vasc Surg. (2022) 85:57–67. 10.1016/j.avsg.2022.04.021 35472500 PMC9627968

[4] Davila VJ Chang JM Stone WM Fowl RJ Bower TC Hinni ML. Current surgical management of carotid body tumors. J Vasc Surg. (2016) 64:1703–10. 10.1016/j.jvs.2016.05.076 27871494

[5] Gonzalez-Urquijo M Castro-Varela A Barrios-Ruiz A Hinojosa-Gonzalez DE Salas AKG Morales EA. Current trends in carotid body tumors: comprehensive review. Head Neck. (2022) 44:2316–32. 10.1002/hed.27147 35838064

[6] Bobadilla-Rosado LO Garcia-Alva R Anaya-Ayala JE Peralta-Vazquez C Hernandez-Sotelo K Luna L. Surgical management of bilateral carotid body Tumors. Ann Vasc Surg. (2019) 57:187–93. 10.1016/j.avsg.2018.10.019 30684613

[7] Kakkos SK Zampakis P Lampropoulos GC Paridis L Kaplanis C Bantouna D. Successful resection of a large carotid body tumor masquerading complete encasement of the internal carotid artery on preoperative imaging. Vasc Endovascular Surg. (2018) 52:304–8. 10.1177/1538574418761722 29495959

[8] Li X Zhang W Shu C Li Q Zhang L Zhu J. Diagnosis and outcomes of surgical treatment of carotid bifurcation tumors. J Int Med Res. (2020) 48:1–12. 10.1177/0300060520976495 33317387 PMC7739102

[9] West CT Brassett C Gaunt ME. Variations in carotid sinus anatomy and their relevance to carotid interventions. Folia Morphol. (2018) 77:693–7. 10.5603/FM.a2018.0017 29500893

[10] Brignole M Moya A de Lange FJ Deharo JC Elliott PM Fanciulli A. 2018 ESC Guidelines for the diagnosis and management of syncope. Eur Heart J. (2018) 39:1883–948. 10.1093/eurheartj/ehy037 29562304

[11] Amin V Pavri BB. Carotid sinus syndrome. Cardiol Rev. (2015) 23:130–4. 10.1097/CRD.0000000000000041 25211534

[12] McIntosh SJ Lawson J Kenny RA. Clinical characteristics of vasodepressor, cardioinhibitory, and mixed carotid sinus syndrome in the elderly. Am J Med. (1993) 95:203–8. 10.1016/0002-9343(93)90261-m 8356984

[13] Truong AT Sturgis EM Rozner MA Truong DT. Recurrent episodes of asystole from carotid sinus hypersensitivity triggered by positioning for head and neck surgery. Head Neck. (2013) 35:E28–30. 10.1002/hed.21812 21739521

[14] Chung HS Park CM Kim ES Ghil BG Park CS. Temporary cardiac arrest in patient under robotically assisted total thyroidectomy causing carotid sinus hypersensitivity -a case report. Korean J Anesthesiol. (2010) 59(Suppl.):S137–140. 10.4097/kjae.2010.59.S.S137 21286424 PMC3030020

[15] Kirchheim HR. Systemic arterial baroreceptor reflexes. Physiol Rev. (1976) 56:100–77. 10.1152/physrev.1976.56.1.100 174143

[16] Hallett JW Jr Nora JD Hollier LH Cherry KJ Jr Pairolero PC. Trends in neurovascular complications of surgical management for carotid body and cervical paragangliomas: a fifty-year experience with 153 tumors. J Vasc Surg. (1988) 7:284–91. 2828696

[17] Casarim AL Tincani AJ Del Negro A Aguiar CG Fanni RV Martins AS. Carotid body tumor: retrospective analysis on 22 patients. São Pãulo Med J. (2014) 132:133–9. 10.1590/1516-3180.2014.1323452 24760216 PMC10852084

[18] Gottlieb A Satariano-Hayden P Schoenwald P Ryckman J Piedmonte M. The effects of carotid sinus nerve blockade on hemodynamic stability after carotid endarterectomy. J Cardiothorac Vasc Anesth. (1997) 11:67–71. 10.1016/s1053-0770(97)90256-1 9058224

[19] Lilitsis E Papaioannou A Hatzimichali A Spyridakis K Xenaki S Chalkiadakis G. A case of asystole from carotid sinus hypersensitivity during patient positioning for thyroidectomy. BMC Anesthesiol. (2016) 16:85. 10.1186/s12871-016-0255-5 27716078 PMC5052875

[20] Lacerda Gde C Pedrosa RC Lacerda RC Santos MC Perez Mde A Teixeira AB. Cardioinhibitory carotid sinus hypersensitivity: prevalence and predictors in 502 outpatients. Arq Bras Cardiol. (2008) 90:148–55. 10.1590/s0066-782x2008000300002 18392392

[21] Bauer AM Smith RB Thorell WE. Implications of carotid sinus hypersensitivity following preoperative embolization of a carotid body tumor. An indication for prophylactic intraoperative cardiac pacing. JAMA Otolaryngol Head Neck Surg. (2014) 140:459–63. 10.1001/jamaoto.2014.144 24651937

[22] da Gama AD Cabral GM. Carotid body tumor presenting with carotid sinus syndrome. J Vasc Surg. (2010) 52:1668–70. 10.1016/j.jvs.2010.07.016 20864295

